# Effect of high density lipoprotein cholesterol (HDL-C) on renal outcome in patients with nephrotic syndrome complicated with steroid-induced diabetes mellitus(SIDM)

**DOI:** 10.1186/s12882-022-03042-9

**Published:** 2023-01-03

**Authors:** Jiarong Li, Di Hui, Liu Yang, Jinhua Hou, Honglang Xie

**Affiliations:** National Clinical Research Center of Kidney Diseases, Jinling Hospital, Nanjing Univerisity School of Medicine, Nanjing, 210016 China

**Keywords:** Idiopathic nephrotic syndrome, Steroid-induced diabetes mellitus, Glucocorticoid, Renal outcome, High density lipoprotein cholesterol

## Abstract

**Objective:**

We aimed to investigate the renal prognosis of patients with idiopathic nephrotic syndrome (INS) complicated with steroid-induced diabetes mellitus (SIDM), the association of high-density lipoprotein cholesterol (HDL-C) before glucocorticoid treatment with renal prognosis, and the risk for persistent diabetes among patients with INS who had withdrawn from steroid therapy.

**Materials and methods:**

We retrospectively analyzed 239 patients with INS complicated with SIDM at the National Clinical Research Center of Kidney Diseases, Jinling Hospital, from January 2008 to December 2019. The primary endpoint was the composite renal outcome defined as the development of end-stage renal disease (ESRD) or a 50% decrease in estimated glomerular filtration rate (eGFR) for more than 24 months after glucocorticoid withdrawal. The secondary endpoint was persistent diabetes, defined as fulfilling the criteria for diagnosing diabetes or using antidiabetic medications for at least 24 months after glucocorticoid withdrawal.

**Results:**

After glucocorticoid withdrawal for over 24 months, 35 (14.6%) patients reached the composite renal endpoint: end-stage renal disease (*n* = 14) or a 50% decrease in eGFR (*n* = 21). Before glucocorticoid therapy, a level of HDL-C greater than 1.45 mmol/L worsened renal survival in patients with INS complicated with SIDM. The log_10_ the level of HDL-C before glucocorticoid treatment was an independent risk factor for the renal outcome. A prediction model was generated: Hazard ratio (renal outcome) = 0.94 * hypertension before glucocorticoid therapy + 2.29 * log_10_ level of HDL-C before glucocorticoid treatment + 0.90 * the grade of interstitial tubule injury (AUROC, 0.75; 95% CI, 0.63 to 0.87; *P* < 0.01). Meanwhile, a level of fasting plasma glucose (FPG) before glucocorticoid treatment greater than 5.2 mmol/L enhanced the likelihood of persistent diabetes for at least 24 months after glucocorticoid withdrawal.

**Conclusions:**

Increased level of HDL-C before glucocorticoid therapy was independently associated with a higher risk for renal outcome and thus may be useful in the renal prognosis of patients with INS complicated with SIDM.

**Supplementary Information:**

The online version contains supplementary material available at 10.1186/s12882-022-03042-9.

## Introduction

Idiopathic nephrotic syndrome (INS) is diagnosed by typical clinical features: heavy proteinuria (more than 3.5 g/24 h) and hypoalbuminemia (less than 30 g/L), often with peripheral edema and hyperlipidemia, after excluding secondary factors such as autoimmune, infection, metabolism, malignant tumors, and drugs [1]. The population incidence of INS is approximately 1–3 per 100,000 people per year [2]. Compared to that in children [3, 4], the pathological etiology of INS in Chinese adults is attributed to membranous nephropathy (MN) (24.96%), IgA nephropathy (IgAN) (24.09%), minimal change disease (MCD) (10.71%), focal segmental glomerulosclerosis (FSGS) (2.45%), membranous proliferative glomerulonephritis (MPGN) (0.61%), postinfectious GN (0.51%), and C3 glomerulopathy (0.14%) in Chinese adults [5]. Adults with INS experience higher adjusted rates of end-stage renal disease (ESRD), cardiovascular outcomes, and death, with significant variation by underlying etiology in the risk of developing ESRD [6].

Lipid disorders are not only acknowledged as one of the clinical characteristic of INS, but also known to be linked to disturbances in oxidative reactions and play an important role in the progression and complications of INS [7], especially high density lipoprotein cholesterol (HDL-C). HDL-C abnormalities in nephrotic syndrome impaired reverse cholesterol transport, contributed to endothelial dysfunction, oxidative stress, and systemic inflammation and consequently promoted atherosclerosis, and glomerulosclerosis [8, 9], which further affected the renal prognosis of patients with INS.

Glucocorticoids remain the mainstream of treatment for INS after excluding the possible secondary factors and have obtained excellent results in improving survival of both patients and kidney function [10]. However, patients with refractory INS are at high risk of medication-induced glucose metabolism disorders, such as diabetes [11, 12]. The prevalence of steroid-induced diabetes mellitus (SIDM) in INS was 6.2% [13]. At present, there was little study about renal prognosis of patients with INS complicated with SIDM, especially the association between HDL-C and renal prognosis. We aimed to investigate the renal prognosis of patients with INS complicated with SIDM. and the association between high-density lipoprotein cholesterol (HDL-C) before glucocorticoid treatment and renal prognosis.

## Materials and methods

### Study design

The study was a retrospective study designed to investigate (1) the renal prognosis after glucocorticoid withdrawal, (2) the association between HDL-C before glucocorticoid treatment and renal prognosis, and (3) the risk factors for persistent diabetes after glucocorticoid withdrawal in patients with INS complicated with SIDM at the National Clinical Research Center of Kidney Diseases, Jinling Hospital, from January 2008 to December 2019.

### Study participants

Patients were eligible for inclusion if they had a diagnosis of INS based on renal biopsy pathology and clinical manifestations, including IgAN, MCD, FSGS, MN, and MPGN; had a diagnosis of SIDM; and were followed up for over 24 months after glucocorticoid withdrawal.

**Fig. 1:**
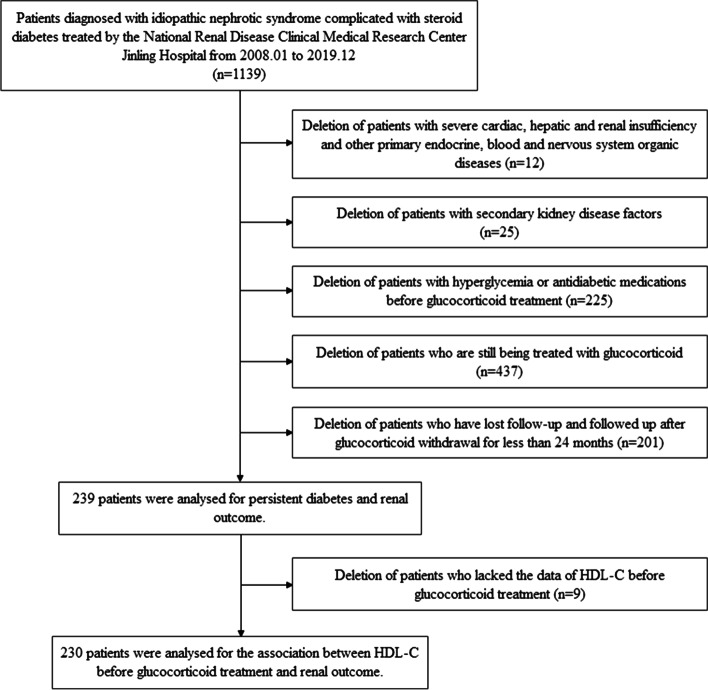
Flow diagram of patient selection

We excluded those who were diagnosed with secondary nephrotic syndrome caused by autoimmune conditions, infection, metabolism, malignant tumors, and drug factors; severe cardiac and hepatic insufficiency; other primary endocrine, neurological, and plasma system organic diseases; and a history of malignant tumors. Exclusion criteria also included age less than 12 years old, hyperglycemia (defined as fasting plasma glucose (FPG) over 7.0 mmol/L and/or postprandial plasma glucose over 11.1 mmol/L) or antidiabetic medications before glucocorticoid treatment, and glucocorticoid treatment without cessation. Patients with ESRD (defined as maintenance dialysis for more than 28 days, kidney transplantation, or estimated glomerular filtration rate (eGFR) < 15 mL/min per 1.73 m^2^ confirmed by a second measurement after at least 28 days) before glucocorticoid withdrawal, patients who were lost to follow-up, and patients with pregnancy, lactation, or who planned to become pregnant were also excluded (Fig. 1).

### Data collection and definitions

The electronic medical records, laboratory results, pathological reports, and medical orders were carefully reviewed to extract the data. INS, including IgAN, MCD, FSGS, MN, and MPGN, was diagnosed according to the Kidney Disease Improving Global Outcomes (KDIGO) clinical practice guidelines. Diabetes was defined as FPG greater than 7.0 mmol/L, or glycosylated hemoglobin (HbA1c) greater than 6.5% or regular hypoglycemic medication. SIDM was defined as an abnormal increase in blood glucose associated with the use of glucocorticoids in a patient without a prior history of diabetes mellitus. The initial course of kidney disease was defined as the interval between the onset of renal injury to the appearance of related symptoms and an abnormal laboratory examination and the initiation of glucocorticoid therapy. The cumulative dose of glucocorticoids was defined as the total amount of glucocorticoid administered from the start of glucocorticoid treatment to the end of treatment (converted to prednisone). The overall course of glucocorticoid treatment was defined as the total time between the beginning of glucocorticoid treatment and its complete discontinuation. Triglyceride-glucose index (TyG index) = In (triglyceride*FPG/2) (unit: mg/d/dL) [14].

Three categories were utilized to classify nephrotic syndrome remission: complete remission (CR), partial remission (PR), and nonremission (NR). CR was defined as the disappearance of the primary clinical symptoms and signs, the absence of urine protein in three consecutive assessments, a urinary protein rate (UPR) less than 0.3 g/24 h, and albumin levels greater than 35 g/L. PR was defined as the disappearance of the majority of clinical symptoms and signs, a decrease in urine protein of more than 50% relative to baseline, a UPR between 0.3 and 3.5 g/24 h, and albumin levels greater than 30 g/L. NR was defined as failing to meet the CR or PR criterion.

### Outcomes

The primary endpoint was the composite renal outcome defined defined as the development of ESRD or a 50% decrease in eGFR (compared to baseline at renal biopsy) [15, 16].

The secondary endpoint was persistent diabetes, which was defined as fulfilling the criteria for diagnosing diabetes or using antidiabetic medications for at least 24 months after glucocorticoid withdrawal.

### Statistical analysis

Categorical variables are described as frequencies and percentages, and continuous variables are described using mean, median, and interquartile range (IQR) values. When the data were normally distributed, independent t tests were used to compare the means of continuous variables. Otherwise, the Mann–Whitney test was used, and the chi-squared test or Fisher’s exact test was used to compare the proportions of categorical variables.We included 2 patients who reached ESRD and 2 patients with a 50% decrease in eGFR within 24 months in the secondary endpoint analysis.

To analyze the composite renal outcome, patients included were followed up at least 24 months or reached the composite renal outcome within 24 months after glucocorticoid withdrawal. We included 2 patients who reached ESRD and 2 patients with a 50% decrease in eGFR within 24 months (these 4 patients followed over 24 months afer glucocorticoid withdrawal) in the secondary endpoint analysis. Logistic regression analyses were used to identify the potential risk factors of persistent diabetes. Survival curves were generated with the Kaplan–Meier method. Cox proportional hazards models were used to identify the potential risk factors of renal outcome. Using receiver operating characteristic curve (ROC) analysis, the area under the curve (AUC) was determined. A ROC curve was performed to evaluate the Cox proportional hazards model prediction value for renal outcome. In addition, an interaction test was conducted to evaluate whether patients’ characteristics influence the relationship between HDL-C before glucocorticoid treatment and renal outcome. *P* < 0.05 denoted statistical significance while using SPSS for Windows version 22 to examine all of the data.

## Results

### Characteristics of patients before glucocorticoid treatment

This study included 239 patients with INS who were diagnosed as SIDM and followed up for more than 24 months after glucocorticoid withdrawal. The median age of the patients was 49 years old (IQR, 39.0–58.0). There were 114 (47.7%) females and 125 males. All patients had normal glucose levels before glucocorticoid treatment. Twenty patients had a family history of diabetes. The average BMI of 239 patients was 24.5 kg/m2 (SD 3.4), the median eGFR was 91.8 mL/min per 1.73 m^2^ (IQR, 65.0–111.8), and the median UPR was 2.8 g/24 h (IQR, 1.4–5.9) before glucocorticoid treatment. Among 239 patients, 128 (53.6%) had MN, 49 (20.5%) had IgAN, 32 (13.4%) had MCD, 27 (11.3%) had FSGS, and 3 (1.3%) had MPGN. The median cumulative dose of glucocorticoids was 8700 mg (IQR, 6300–12,900). The total course of glucocorticoid treatment was 20 months (IQR, 12.0–31.0). The median diagnosis time of SIDM was 2.0 months (IQR, 1.0–5.0). During follow-up, 32 (13.4%) patients had new-onset hypertension, 4 (1.7%) patients had osteonecrosis of the femoral head (Supplement 1).

### Glucose metabolism outcome

After glucocorticoid withdrawal for at least 24 months, 65 patients still satisfied the diagnostic criteria for diabetes mellitus without antidiabetic medications (*n* = 26) or still took antidiabetic medications (*n* = 39). One hundred thirty-six individuals had normal glucose metabolism without antidiabetic medications, while 38 patients had impaired glucose tolerance.

The distribution of age and gender did not differ significantly with or without persistent diabetes. Compared to patients without persistent diabetes, patients with persistent diabetes had higher FPG, a higher TyG index, and a higher proportion of cyclophosphamidebefore glucocorticoid treatment. The distribution of pathogenic categories varied considerably among 239 individuals (*P* = 0.03). Among patients with persistent diabetes, 35 (53.8%) had MN, 9 (13.8%) had IgAN, 9 (13.8%) had MCD, 9 (13.8%) had FSGS, and 3 (4.6%) had MPGN, whereas in the group of patients without persistent diabetes, 93 (53.4%) had MN, 40 (23.0%) had IgAN, 23 (13.2%) had MCD, 18 (10.3%) had FSGS, and none had MPGN. The incidence of tubular atrophy, interstitial inflammation, interstitial fibrosis grade and interstitial tubule injury were similar among patients with or without persistent diabetes. The cumulative dose and the total course of glucocorticoid treatment were similar between those with and without persistent diabetes. There was no significant difference among patients with and without persistent diabetes for osteonecrosis of the femoral head, during the follow-up period. However, patients with persistent diabetes have high proportion of new hypertension during the follow-up period (Supplement 1).

FPG before glucocorticoid treatment was the only independent risk factor for persistent diabetes (OR, 1.98; 95% CI, 1.03 to 3.80; *P* = 0.04); while the cumulative dose and total course of glucocorticoid treatment were not the risk factors for the outcome. Based on the analysis of the Roc curve, FPG > 5.2 mmol/L before glucocorticoid treatment increased the risk for the persistent diabetes outcome (Table 1).

**Table 1:** Risk factors for persistent diabetes in patients with INS complicated with SIDM

Factor	Single factor analysis		Multiple factor analysis	
	OR(95%CI)	*P*	OR(95%CI)	*P*
BMI	1.096(1.007~1.192)	0.033	1.080(0.988~1.181)	0.092
The time of SIDM diagnosis	1.019(0.997~1.043)	0.092	1.018(0.992~1.044)	0.188
FPG	1.802(1.078~3.012)	0.025	1.978(1.030~3.796)	0.040
TG	1.217(1.009~1.468)	0.040	1.457(0.755~2.812)	0.262
TG/HDL	1.255(1.030~1.528)	0.024	1.194(0.863~1.652)	0.285
TyG index	1.725(1.007~2.954)	0.047	0.375(0.061~2.311)	0.290

*BMI* Body Mass Index, *FPG* Fasting Plasma Glucose, *SIDM* steroid-induced diabetes mellitus, *TG* Triglycerides, *HDL- C* High-Density Lipoprotein Cholesterol, *TyG index *Triglyceride-glucose index = In (Triglyceride * Fasting blood glucose / 2) (unit: mg/d/dL)

### Renal outcome

The median follow-up time from renal biopsy of 239 patients was 92 months (IQR, 63.0–109.0) and the median follow-up time after glucocorticoid withdrawal was 61 months (IQR, 33.0–84.0). After glucocorticoid withdrawal for more than 24 months, 35 patients reached composite endpoints: ESRD (*n* = 14) or a 50% decrease in eGFR (*n* = 21) during the follow-up. Ten patients with persistent diabetes reached composite endpoints: ESRD (*n* = 3) or a 50% decrease in eGFR (*n* = 7), and 25 patients without persistent diabetes reached composite endpoints: ESRD (*n* = 11) or more than 50% irreversible decrease in the eGFR level (*n* = 14). There was no significant difference between those with and without persistent diabetes in composite endpoints.

### Association between HDL-C before glucocorticoid treatment and renal outcome

Nine patients were excluded from the analysis for lacking the information of HDL-C. The median level of HDL-C before glucocorticoid treatment of the remained 230 patients was 1.45 mmol/L (1.07 to 1.93), and all patients were divided into a high level HDL-C group and a low level HDL-C group according to the median HDL-C level. There was no significant difference in age, sex, BMI, family history of diabetes or incidence of basic hypertension between the two groups. There was no significant difference in the initial course of renal disease or the incidence of INS complications, including AKI, infection and thrombosis, between the two groups. The distribution of pathological types was similar between the two groups. The incidence of tubular atrophy, interstitial inflammation, the grade of interstitial fibrosis and interstitial tubule injury were similar between the two groups. The proportion of patients who had been treated with cyclophosphamide before glucocorticoid treatment in high level of HDL-C group was higher. There was no significant difference in the induced dose and course of glucocorticoid treatment or the total dose and course of glucocorticoid treatment between the two groups. The incidence of new hypertension was higher in the high level of HDL-C group, but there was no significant difference in the incidence of osteonecrosis of the femoral head between the two groups (Table 2). During the follow-up, patients with HDL-C over 1.45 mmol/L and no more than 1.45 mmol/L had similar relapses times and mean annual eGFR decline rate (Supplement 2).

**Table 2:** Baseline Characteristics of participants in a study of effect of HDL-C on renal outcome in patients with INS complicated with SIDM

Characteristics	No. ^**a**^			***P*** Value
	Total (***n*** = 230)	Low level of HDL-C (***n*** = 117)	High level of HDL-C (***n*** = 113)	
**Sex**				
Female	109 (47.4)	50 (42.7)	59 (52.2)	0.150
Male	121 (52.6)	67 (57.3)	54 (47.8)	0.150
Age, y	49.0 (39.0, 58.0)	48.0 (40.0, 58.0)	50.0 (38.0, 57.5)	0.621
Family history of diabetes mellitus	18 (8.0)	7 (6.1)	11 (9.9)	0.289
Baseline hypertension	69 (30.0)	41 (35.0)	28 (24.8)	0.089
Time from abnormality, mo	4.0 (1.5, 10.0)	4.0 (1.5, 10.0)	3.0 (1.8, 9.5)	0.868
**Nephrotic syndrome complications**				
AKI	22 (9.6)	12 (10.3)	10 (8.8)	0.717
Infection	31 (13.5)	14 (12.0)	17 (15.0)	0.494
Thrombosis	9 (3.9)	4 (3.4)	5 (4.4)	0.694
**Laboratory findings**				
FPG, mmol/L	5.1 ± 0.6	5.1 ± 0.6	5.1 ± 0.6	0.961
TG, mmol/L	2.5 (1.7, 3.4)	2.6 (1.7, 3.5)	2.3 (1.7, 3.4)	0.550
TyG index^b^,	9.2 ± 0.5	9.2 ± 0.5	9.2 ± 0.6	0.768
SCr, mg/dl	0.8 (0.7, 1.1)	0.8 (0.7, 1.1)	0.8 (0.7, 1.0)	0.333
eGFR, ml/min per 1.73m^2^	91.9 (65.3, 111.7)	94.5 (67.7, 113.9)	91.4 (64.9, 107.0)	0.794
UA, μmol/L	377.7 ± 110.3	390.8 ± 109.2	364.1 ± 110.2	0.066
Alb, mean ± SD, g/L	32.6 ± 7.9	33.4 ± 8.5	31.7 ± 7.0	0.100
UPR, g/24 h	2.9 (1.4, 6.1)	2.3 (1.0, 5.2)	3.6 (1.9, 6.6)	0.004
**Pathological classification**				0.120
MN	121 (52.6)	55 (47.0)	66 (58.4)	
IgAN	47 (20.4)	27 (23.1)	20 (17.7)	
FSGS	27 (11.7)	12 (10.3)	15 (13.3)	
MCD	32 (13.9)	20 (17.1)	12 (10.6)	
MPGN	3 (1.3)	3 (2.6)	0 (0.0)	
**Glomerulus injury**				
Glomerulus sclerosis	4.8 (0.0, 14.3)	5.7 (0.0, 15.2)	4.5 (0.0, 12.5)	0.430
Focal sclerosis	0.0 (0.0, 7.2)	0.0 (0.0, 6.7)	0.0 (0.0, 9.1)	0.327
Crescent	0.0 (0.0, 0.0)	0.0 (0.0, 0.0)	0.0 (0.0, 0.0)	0.262
**Tubulointerstitial injury**				
Tubular atrophy	140 (60.9)	77 (65.8)	63 (55.8)	0.118
Intestinal inflammation	173 (75.2)	91 (77.8)	82 (72.6)	0.360
**Interstitial fibrosis**^**c**^				0.563
Grade 1	139 (60.4)	67 (57.3)	72 (63.7)	
Grade 2	67 (29.1)	36 (30.8)	31 (27.4)	
Grade 3	24 (10.4)	14 (12.0)	10 (8.8)	
**Tubulointerstitial damage**^**d**^				0.381
Grade 1	194 (84.3)	97 (82.9)	97 (85.8)	
Grade 2	28 (12.2)	14 (12.0)	14 (12.4)	
Grade 3	8 (3.5)	6 (5.1)	2 (1.8)	
**Glucocorticoid medication**				
The induction dose, mg/d	60.0 (50.0, 60.0)	60.0 (50.0, 60.0)	60.0 (47.5, 60.0)	0.386
The total dose, mg	8.7 (6.3, 12.8)	8.8 (5.5, 12.8)	8.7 (6.3, 13.1)	0.989
The induction course, mo	1.5 (1.0, 2.0)	1.5 (1.0, 2.0)	1.5 (1.0, 2.0)	0.385
The total course, mo	20.0 (12.0, 31.0)	20.0 (11.0, 30.0)	20.0 (13.0, 33.0)	0.685
Time till SDM diagnosis, mo	2.3 (1.0, 5.0)	2.5 (1.0, 5.0)	2.0 (1.0, 5.0)	0.998
**Glucocorticoid complications**				
new hypertension	32 (13.9)	10 (8.5)	22 (19.5)	0.017
osteonecrosis of femoral head	4 (1.7)	2 (1.7)	2 (1.8)	0.972
persistent diabetes outcome	61 (26.5)	34 (29.1)	27 (23.9)	0.375
**Medication before glucocorticoid**				
Tripterygium	23 (10.0)	12 (10.3)	11 (9.7)	0.895
Cyclophosphamide	8 (3.5)	0 (0.0)	8 (7.1)	0.003
Tacrolimus	2 (0.9)	1 (0.9)	1 (0.9)	0.980
Mycophenolate mofetil	1 (0.4)	0 (0.0)	1 (0.9)	0.308
Statins	86 (37.4)	38 (32.5)	48 (42.5)	0.117
ACEI/ARB	120 (52.2)	63 (53.8)	57 (50.4)	0.605
Other antihypertension drugs	39 (17.0)	23 (19.7)	16 (14.2)	0.267

^a^Percentages may not total 100 because of rounding; ^b^TyG index: Triglyceride-glucose index = In (Triglyceride * Fasting blood glucose / 2) (unit: mg/d/dL); ^c^Interstitial fibrosis grade: grade 1 (lesion range less than 25%); grade 2 (lesion range 25 to 50%); grade 3 (lesion range more than 50%); ^d^Tubulointerstitial damage grade: grade 1 (lesion range less than 25%); grade 2 (lesion range 25 to 50%); grade 3 (lesion range more than 50%). *BMI* Body mass index, *FPG* Fasting plasma glucose, *HDL-C* High-density lipoprotein cholesterol, *INS* Idiopathic nephrotic syndrome, *SIDM* Steroid-induced diabetes mellitus, *TG* Triglycerides, *SCr* Serum creatinine, *eGFR* Glomerular filtration rate estimated according to CKD-EPI Formula, *Alb* Albumin, *UPR *Urinary protein rate, *MN* Membranous nephropathy, *IgAN* IgA nephropathy, *FSGS* Focal segmental glomerulosclerosis, *MCD* Minimally pathological nephropathy

**Fig. 2:**
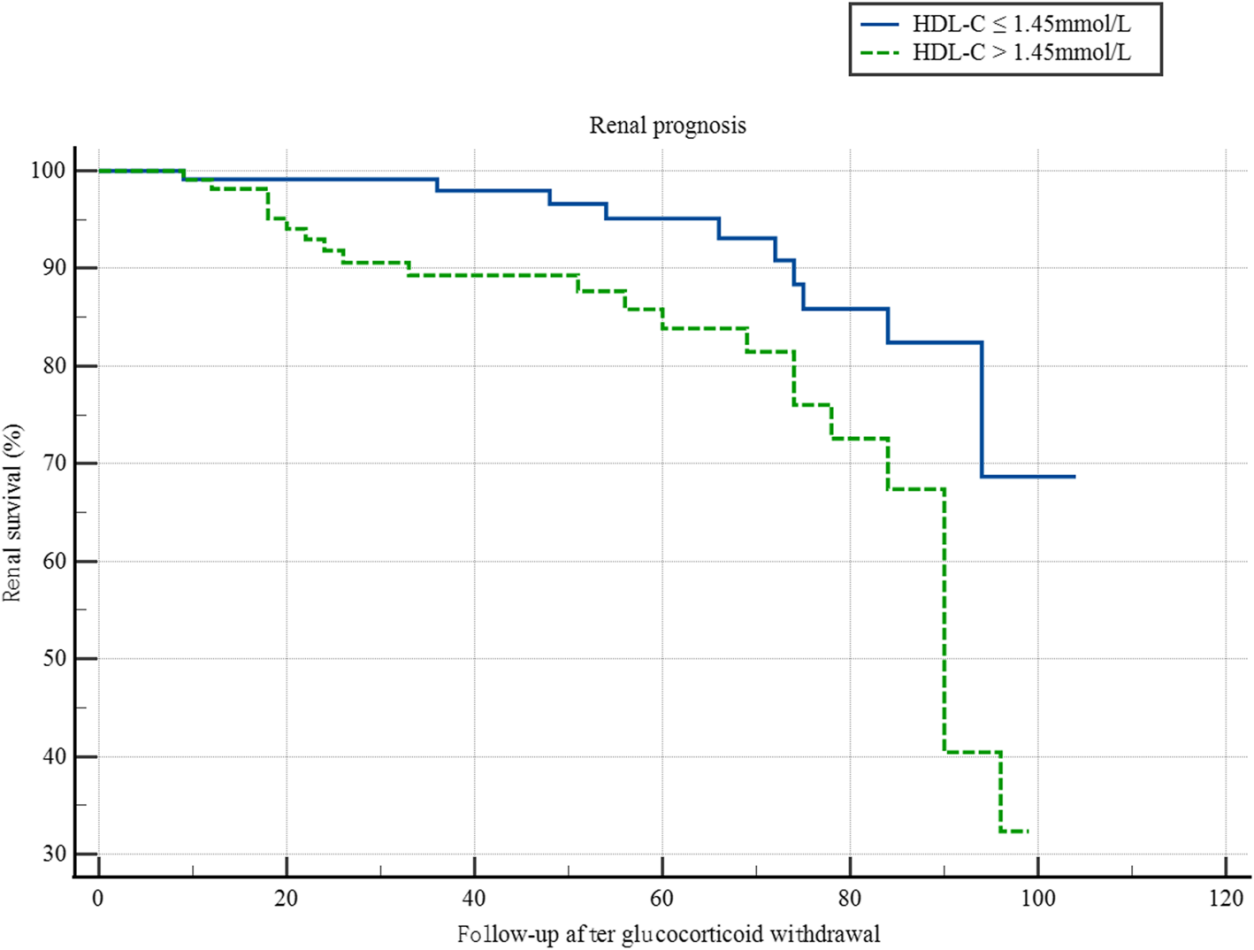
Kaplan–Meier analysis of renal outcome in patients with the level of HDL-C. Patients with the level of HDL-C more than 1.45 mmol/L before glucocorticoid treatment were tend to have poor renal outcome

One patient with ESRD lacked HDL-C information before glucocorticoid treatment Eleven patients whose HDL-C was no more than 1.45 mmol/L reached composite endpoints: ESRD (*n* = 4) or a 50% decrease in eGFR (*n* = 7), and 23 patients whose HDL-C was more than 1.45 mmol/L reached composite endpoints: ESRD (*n* = 9) or a 50% decrease in eGFR (*n* = 14). HDL-C levels before glucocorticoid therapy more than 1.45 mmol/L were associated with higher risk of renal outcome according to Kaplan-Meier analyses (Fig. 2).

**Fig. 3:**
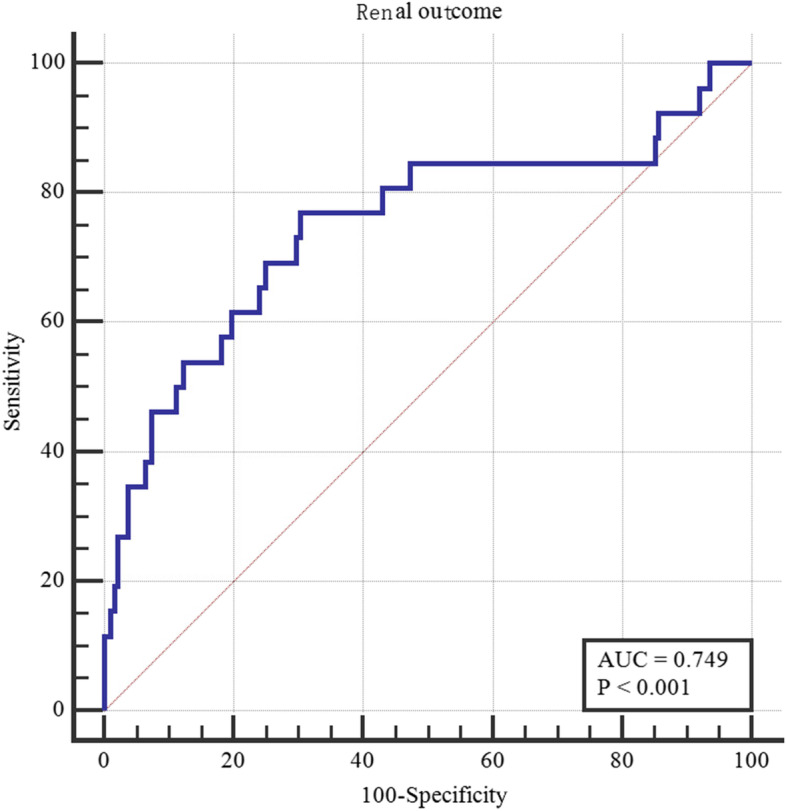
Roc curve for renal outcome based on cox proportional hazards model. A prediction model for renal prognosis included the factors associated with renal outcome, such as the log10 level of HDL-C before glucocorticoid treatment, hypertension before glucocorticoid therapy, and the grade of interstitial tubule injury was constructed. Hazard ratio (renal outcome) = 0.94 * hypertension before glucocorticoid therapy + 2.29 * log10 level of HDL-C before glucocorticoid treatment + 0.90 * the grade of interstitial tubule injury. The hazard ratio model identified patients who reached the composite renal endpoints. (AUROC, 0.75; 95% CI, 0.63 to 0.87; *P* < 0.01)

In adjusted Cox proportional hazards models, the log10 level of HDL-C before glucocorticoid treatment (HR, 9.85; 95% CI, 1.36 to 71.16; *P* = 0.02), hypertension before glucocorticoid therapy (HR, 2.56; 95% CI, 1.01 to 6.52; *P <* 0.05), and the grade of interstitial tubule injury (HR, 2.46; 95% CI, 1.33 to 4.55; *P* < 0.01) were associated with an increased risk of renal outcome (Table 3). This study constructed a prediction model for renal prognosis included the factors associated with renal outcome, such as the log_10_ level of HDL-C before glucocorticoid treatment, hypertension before glucocorticoid therapy, and the grade of interstitial tubule injury. Hazard ratio (renal outcome) = 0.94 * hypertension before glucocorticoid therapy + 2.29 * log_10_ level of HDL-C before glucocorticoid treatment + 0.90 * the grade of interstitial tubule injury. The hazard ratio model identified patients who reached the composite renal endpoints. (AUROC, 0.75; 95% CI, 0.63 to 0.87; *P* < 0.01) (Fig. 3).

**Table 3:** Risk factors of renal outcome in a study of effect of HDL-C on renal outcome in patients with INS complicated with SIDM

Factor	Single factor analysis		Multiple factor analysis	
	HR (95%CI)	P	HR (95%CI)	P
Age	1.05 (1.02 to 1.08)	0.001	1.02 (0.99 to 1.05)	0.201
Hypertension before Glucocorticoid treatment	3.54 (1.77 to 7.08)	< 0.001	2.56 (1.01 to 6.52)	0.048
Infection	2.18 (1.01 to 4.69)	0.046	1.04 (0.34 to 3.15)	0.951
Focal sclerosis	1.03 (1.00 to 1.06)	0.034	1.01 (0.98 to 1.04)	0.536
Tubulointerstitial damage	2.24 (1.32 to 3.83)	0.003	2.46 (1.33 to 4.55)	0.004
Log_10_HDL-C	6.48 (1.57 to 26.78)	0.010	9.85 (1.36 to 71.16)	0.023

*HDL-C* High density lipoprotein cholesterol, *INS* Idiopathic nephrotic syndrome, *SIDM* Steroid-induced diabetes mellitus

### Subgroup analysis

In this study, the relationship between the level of HDL-C before glucocorticoid treatment and renal prognosis was further stratified by age, baseline hypertension, infection, focal sclerosis, and at least Grade 1 tubulointerstitial damage. The results suggested that age, focal sclerosis, and at least Grade 1 tubulointerstitial damage were considered the prominent interactive factors that affect the association between HDL before glucocorticoid treatment and the risk of renal prognosis by the interaction analysis, which remained robust under the grouping of other indicators (*P* < 0.05) (Table 4).

**Table 4:** Subgroup analysis of renal outcome in a study of effect of HDL-C on renal outcome in patients with INS complicated with SIDM

Subgroup	Cases	HR (95%CI)	*P* value	*P* for interaction
Age (years)				0.022
>60y	18/52	1.41 (0.89,2.24)	0.139	
≤60y	16/178	1.13 (0.79,1.60)	0.503	
Baseline hypertension				0.055
Yes	21/69	1.55 (0.99,2.41)	0.054	
No	13/161	1.18 (0.84,1.68)	0.343	
Infection				0.862
Yes	9/31	1.16 (0.61,2.23)	0.650	
No	25/199	1.17 (0.88,1.56)	0.273	
Focal sclerosis(%)				0.002
> 12	19/52	1.45 (0.92,2.29)	0.108	
≤12	15/176	1.05 (0.66,1.69)	0.829	
At least Grade 1 tubulointerstitial damage				0.002
Yes	11/36	1.77 (0.79,3.97)	0.169	
No	23/194	1.20 (0.90,1.58)	0.213	

*HDL-C* High density lipoprotein cholesterol, *INS* Idiopathic nephrotic syndrome, *SIDM* Steroid-induced diabetes mellitus

## Discussion

The key finding from these retrospective analyses in patients with INS complicated with SIDM was that the level of HDL-C before glucocorticoid treatment as an independent risk factor had significant prognostic value for renal outcome. Before glucocorticoid therapy, the level of HDL-C more than 1.45 mmol/L affected the renal survival in patients with INS complicated with SIDM. Meanwhile, this research provided a simple and efficient method for evaluating long-term risk for renal prognosis in patients with INS complicated with SIDM. In addition, FPG > 5.2 mmol/L prior to glucocorticoid treatment enhanced the likelihood of persistent diabetes.

### Glucose metabolism outcome

Previous studies showed that fasting hyperglycemia and abnormal HbA1c should be taken seriously, monitoring FPG and HbA1c regularly is required in people with secondary factors for hyperglycemia. To begin with, Thamakaison showed fasting hyperglycemia could enhance the evolution of diabetes with deteriorating diabetes, and cumulative exposure to poor FPG would increase the future risk of type 2 diabetes dose-dependently [17]. Further studies found that patients with both impaired fasting glucose and abnormal HbA1c had a greater chance of developing diabetes than patients with impaired fasting glucose alone [18]. Moreover, patients with refractory nephrotic syndrome had an increased risk of glucose metabolism disorders, regardless of their weight status [19].

### Mechanism of HDL abnormalities in INS

The following points briefly describe the mechanisms of HDL structural and functional abnormalities in INS [9]. First, HDL-mediated extraction of cholesterol from lipid-laden macrophages and mesangial and other cell types is limited due to increased expression of acyl-CoA cholesterol acyltransferase-1 (ACAT-1) in vascular and renal tissue, severe urinary loss, and marked reduction in serum lecithin cholesteryl ester acyltransferase (LCAT) levels [8]. In addition, serum cholesteryl ester transfer protein (CETP) was significantly enhanced, resulting in additional HDL-C depletion and increased triglyceride content [20]. Furthermore, by inducing downregulation of PDZ-containing kidney protein 1 (PDZK1), [21], INS [22] leads to a marked decrease in the hepatic HDL docking receptor (scavenger receptor class B, type 1 [SR-B1]) [23], which is the gateway for loading HDL-C and hepatic lipase-mediated extraction of triglycerides and phospholipids.

### Association between HDL-C and renal outcome

According to existing research findings, age and mean proteinuria during follow-up were independent risk factors for poor renal prognosis, in addition to proteinuria, hypertension or impaired renal function at baseline and crescents involving up to 75% of glomeruli [24], which is different from our previous understanding. This study is the first to suggest a relationship between HDL-C before glucocorticoid treatment and renal prognosis in patients with INS.

In individuals with chronic kidney diseases (CKD), HDL insufficiency and dysfunction contribute to the progression of renal disease [25, 26]. Normal HDL maintains endothelial function and nitric oxide production, whereas HDL deficiency and dysfunction aggravate the severity of oxidative stress and inflammatory cell infiltration in renal tissue and further contribute to the progression of renal disease [27]. Most importantly, the proximal tubules in patients and animal models of proteinuria reabsorb filtered lipid-carrying proteins through the glomerulus, leading to lipid accumulation in the proximal tubule epithelial cells, promoting glomerulosclerosis tubular damage and dysfunction, and CKD progression [28].

In the clinic, there are many studies on the association between HDL-C and the progression of renal disease in patients with CKD [26]. It is believed that HDL-C levels that are too high or too low will be higher risk for the progression of CKD. A study of 1,943,682 male veterans found a U-shaped association between HDL-C and reduced eGFR and ESRD. Subjects in the lowest and highest deciles of HDL-C were associated with an increased risk of CKD progression [29]. In the general middle-aged nondiabetic population, higher HDL-C levels (> 1.6 mmol/L) were independently associated with rapid loss of eGFR. Elevated HDL-C levels are associated with an increased risk of sharp declines in GFR and rapid declines in GFR [30]. In contrast to high HDL-C, a low serum HDL-C level (0.99 ± 0.14 mmol/L) was significantly associated with a greater than 30% decrease in eGFR or ESRD (HR 4.80, *P* < 0.01). In female patients with CKD under the age of 70 years, low serum HDL-C levels are a significant predictor of CKD progression [31]. Furthermore, in the Chinese elderly population, renal insufficiency was independently associated with low HDL-C, and even slight changes in renal function may be associated with altered lipid levels [32].

### Limitations

This study had several limitations. First, as a retrospective study, not all patients were monitored for 2-hour postprandial blood glucose and HbA1c. Second, as a result of the limitations of the retrospective design, we were unable to monitor the level of HDL-C during follow-up. In addition, as a single-center study with a limited sample size, this research had limited generalizability.

## Conclusion

In summary, this study demonstrated that the level of HDL-C prior to glucocorticoid therapy was independently associated with a worse renal prognosis in patients with INS complicated with SIDM. The elevated level of HDL-C is a simple and efficient marker to evaluate the long-term risk in patients with INS complicated with SIDM.

## Supplementary Information

**Additional file 1: Supplement 1.** Baseline Characteristics of participants in patients with INS complicated with SIDM.

**Additional file 2: Supplement 2.** Follow-up characteristics of participants in a study of effect of HDL-C on renal outcome in patients with INS complicated with SIDM.

## Data Availability

The information and data of the study population were extracted from Hospital Information System. The datasets are not publicly available because the individual privacy of the participants should be protected. Data are however available from the corresponding author on reasonable request.

## Abbreviations

*ACAT-1*: Acyl-CoA cholesterol acyltransferase-1
BMI: Body mass index
CETP: Cholesteryl ester transfer protein
CKD: Chronic kidney diseases
CR: Complete remission
FPG: Fasting plasma glucose
FSGS: Focal segmental glomerulosclerosis
HDL: High-density lipoprotein
HDL-C: High-density lipoprotein cholesterol
IgAN: IgA nephropathy
INS: Idiopathic nephrotic syndrome
LCAT: Lecithin cholesteryl ester acyltransferase
LDL-C: Low-density lipoprotein cholesterol
MCD: Minimal pathological nephropathy
MN: Membranous nephropathy
NR: Non-remission
PR: Partial remission
PDZK1: PDZ-containing kidney protein 1
SIDM: Steroid-induced diabetes mellitus
SR-B1: Scavenger receptor class B, type 1
SCr: Serum creatinine
TC: Total cholesterol
TyG index: Triglyceride-glucose index
eGFR: Estimated glomerular filtration rate estimated according to CKD-EPI formula
UPR: Urinary protein rate
