# Supplementary material for: Effect of high density lipoprotein cholesterol (HDL-C) on renal outcome in patients with nephrotic syndrome complicated with steroid-induced diabetes mellitus(SIDM)
Source: BMC Nephrol. 2023 Jan 3;24:2. doi: 10.1186/s12882-022-03042-9 (PMC9809113; doi:10.1186/s12882-022-03042-9)
Supplement: Supplementary file 1 — Additional file 1: Supplement 1. Baseline Characteristics of participants in patients with INS complicated with SIDM. [file 12882_2022_3042_MOESM1_ESM.docx]

**Supplement 1. Baseline Characteristics of participants in patients with INS complicated with SIDM.**

| **Characteristics** | **No. ^a^** | | | **P Value** |
| --- | --- | --- | --- | --- |
|  | **Total**  **(n=239)** | **Non-DM Group**  **(n=174)** | **DM Group**  **(n=65)** |  |
| **Sex** |  |  |  |  |
| Female | 114 (47.7) | 80 (46.2) | 34 (52.3) | 0.383 |
| Male | 125 (52.3) | 94 (53.8) | 31 (47.7) | 0.383 |
| Age, y | 49.0 (39.0,58.0) | 48.0 (40.0,56.2) | 51.0 (35.5,60.0) | 0.406 |
| Family history of diabetes mellitus | 20 (8.5) | 12 (6.9) | 8 (12.9) | 0.149 |
| Baseline hypertension | 71 (29.7) | 48 (27.6) | 23 (35.4) | 0.240 |
| Time from abnormality, mo | 4.0 (1.5,10.0) | 3.0 (1.5,9.0) | 5.0 (2.0,10.5) | 0.221 |
| **Nephrotic syndrome complications** |  |  |  |  |
| AKI | 24 (10.0) | 18 (10.3) | 6 (9.2) | 0.799 |
| Infection | 32 (13.4) | 22 (12.6) | 10 (15.4) | 0.539 |
| Thrombosis | 9 (3.8) | 7 (4.0) | 2 (3.1) | 0.732 |
| **Laboratory findings** |  |  |  |  |
| FPG, mmol/L | 5.1 ± 0.6 | 5.0 ± 0.6 | 5.2 ± 0.6 | 0.023 |
| TG, mmol/L | 2.5 (1.7,3.4) | 2.4 (1.7,3.3) | 2.6 (1.7,4.4) | 0.123 |
| HDL-C, mmol/L | 1.5 (1.1,1.9) | 1.5 (1.1,2.0) | 1.3 (1.0,1.7) | 0.146 |
| TyG index^b^, | 9.2 ± 0.5 | 9.1 ± 0.5 | 9.3 ± 0.6 | 0.046 |
| SCr, mg/dl | 0.8 (0.7,1.1) | 0.8 (0.7,1.1) | 0.8 (0.7,1.0) | 0.525 |
| eGFR, ml/min per 1.73m^2^ | 91.8 (65.0,111.8) | 93.5 (69.4,113.1) | 89.2 (64.0,106.2) | 0.298 |
| UA, μmol/L | 377.9 ± 108.7 | 377.4 ± 112.7 | 382.3 ± 100.4 | 0.758 |
| Alb, mean ± SD, g/L | 32.6 ± 7.9 | 32.9 ± 7.9 | 32.0 ± 7.8 | 0.475 |
| UPR, g/24h | 2.8 (1.4,5.9) | 2.8 (1.3,5.6) | 3.4 (1.6,7.0) | 0.325 |

(continued)

| **Characteristics** | **No. ^a^** | | | **P Value** |
| --- | --- | --- | --- | --- |
|  | **Total**  **(n=239)** | **Non-DM Group**  **(n=174)** | **DM Group**  **(n=65)** |  |
| **Pathological classification** |  |  |  | 0.033 |
| MN | 128 (53.6) | 93 (53.4) | 35 (53.8) |  |
| IgAN | 49 (20.5) | 40 (23.0) | 9 (13.8) |  |
| FSGS | 27 (11.3) | 18 (10.3) | 9 (13.8) |  |
| MCD | 32 (13.4) | 23 (13.2) | 9 (13.8) |  |
| MPGN | 3 (1.3) | 0 (0.0) | 3 (4.6) |  |
| **Glomerulus injury** |  |  |  |  |
| Glomerulus sclerosis | 4.8 (0.0,14.3) | 5.1 (0.0,15.3) | 4.0 (0.0,10.1) | 0.376 |
| Focal sclerosis | 0.0 (0.0,7.7) | 0.0 (0.0,8.0) | 0.0 (0.0,5.9) | 0.389 |
| Crescent | 0.0 (0.0,0.0) | 0.0 (0.0,0.0) | 0.0 (0.0,0.0) | 0.675 |
| **Tubulointerstitial injury** |  |  |  |  |
| Tubular atrophy | 149 (62.3) | 110 (63.2) | 39 (60.0) | 0.648 |
| Intestinal inflammation | 182 (76.2) | 133 (76.4) | 49 (75.4) | 0.865 |
| **Interstitial fibrosis^c^** |  |  |  | 0.268 |
| Grade 1 | 142 (59.4) | 105 (60.3) | 37 (56.9) |  |
| Grade 2 | 71 (29.7) | 49 (28.2) | 22 (33.8) |  |
| Grade 3 | 26 (10.9) | 20 (11.5) | 6 (9.2) |  |
| **Tubulointerstitial damage^d^** |  |  |  | 0.600 |
| Grade 1 | 200 (83.7) | 144 (82.8) | 56 (86.2) |  |
| Grade 2 | 30 (12.6) | 24 (13.8) | 6 (9.2) |  |
| Grade 3 | 9 (3.8) | 6 (3.4) | 3 (4.6) |  |

(continued)

| **Characteristics** | **No. ^a^** | | | **P Value** |
| --- | --- | --- | --- | --- |
|  | **Total**  **(n=239)** | **Non-DM Group**  **(n=174)** | **DM Group**  **(n=65)** |  |
| **Glucocorticoid medication** |  |  |  |  |
| The induction dose, mg/d | 60.0 (45.0,60.0) | 60.0 (48.8,60.0) | 60.0 (45.0,60.0) | 0.509 |
| The total dose, g | 8.7 (6.3,12.9) | 8.9 (6.3,12.7) | 8.4 (5.7,13.8) | 0.680 |
| The induction course, mo | 1.5 (1.0,2.0) | 1.5 (1.0,2.0) | 1.8 (1.0,2.0) | 0.447 |
| The total course, mo | 20.0 (12.0,31.0) | 20.0 (12.0,30.0) | 20.0 (12.0,36.2) | 0.406 |
| Time till SDM diagnosis, mo | 2.0 (1.0,5.0) | 2.0 (1.0,4.6) | 3.0 (1.0,7.0) | 0.130 |
| **Glucocorticoid complications** |  |  |  |  |
| new hypertension | 32 (13.4) | 21 (12.1) | 11 (16.9) | 0.327 |
| osteonecrosis of femoral head | 4 (1.7) | 3 (1.7) | 1 (1.5) | 0.921 |
| **Medication before glucocorticoid** |  |  |  |  |
| [Tripterygium](http://www.baidu.com/link?url=w78_K27LfSgr7lLWOcpb9CwAChkR2-1HjNJOkcz01Ro8Pq-742YMi-9wsrPvwFhHT9c_MECMuXzxvbYciWHFljo7rHTfO3s5m1ZBxSKoihG) | 24 (10.0) | 17 (9.8) | 7 (10.8) | 0.819 |
| Cyclophosphamide | 8 (3.3) | 5 (2.9) | 3 (4.6) | 0.505 |
| Tacrolimus | 3 (1.3) | 3 (1.7) | 0 (0.0) | 0.287 |
| Mycophenolate mofetil | 1 (0.4) | 1 (0.6) | 0 (0.0) | 0.540 |
| Statins | 90 (37.7) | 66 (37.9) | 24 (36.9) | 0.886 |
| ACEI/ARB | 127 (53.1) | 84 (48.3) | 43 (66.2) | 0.014 |
| Other antihypertension drugs | 40 (16.7) | 24 (13.8) | 16 (24.6) | 0.046 |

a Percentages may not total 100 because of rounding; b TyG index:Triglyceride-glucose index = In (Triglyceride * Fasting blood glucose / 2) (unit: mg/d/dL); c Interstitial fibrosis grade: grade 1 (lesion range less than 25%); grade 2 (lesion range 25% to 50%); grade 3 (lesion range more than 50%); d Tubulointerstitial damage grade: grade 1 (lesion range less than 25%); grade 2 (lesion range 25% to 50%); grade 3 (lesion range more than 50%).BMI: Body mass index; DM: diabetes mellitus; FPG: Fasting plasma glucose; HDL-C: high-density lipoprotein cholesterol; INS: idiopathic nephrotic syndrome; SIDM: steroid-induced diabetes mellitus; TG: Triglycerides; SCr: Serum creatinine; eGFR: Glomerular filtration rate estimated according to CKD-EPI Formula; Alb: Albumin;UPR:Urinary protein rate; MN: Membranous nephropathy; IgAN: IgA nephropathy; FSGS: Focal segmental glomerulosclerosis; MCD: Minimally pathological nephropathy.
