# Supplementary material for: Effect of high density lipoprotein cholesterol (HDL-C) on renal outcome in patients with nephrotic syndrome complicated with steroid-induced diabetes mellitus(SIDM)
Source: BMC Nephrol. 2023 Jan 3;24:2. doi: 10.1186/s12882-022-03042-9 (PMC9809113; doi:10.1186/s12882-022-03042-9)
Supplement: Supplementary file 2 — Additional file 2: Supplement 2. Follow-up characteristics of participants in a study of effect of HDL-C on renal outcome in patients with INS complicated with SIDM. [file 12882_2022_3042_MOESM2_ESM.docx]

**Supplement 2. Follow-up characteristics of participants in a study of effect of HDL-C on renal outcome in patients with INS complicated with SIDM**

| **Characteristics** | **No. ^a^** | | | | **P Value** | |
| --- | --- | --- | --- | --- | --- | --- |
|  | **Total**  **(n=230)** | | **Low level of HDL-C**  **(n = 117)** | **High level of HDL-C**  **(n = 113)** |  |  |
| **Curative effect** |  | |  |  | 0.248 | |
| NR | 31 (13.5) | | 12 (10.3) | 19 (16.8) |  | |
| PR | 134 (58.3) | | 68 (58.4) | 66 (58.4) |  | |
| CR | 65 (28.3) | | 37 (31.6) | 28 (24.8) |  | |
| Relapse, frequency | 0.0 (0.0,1.0) | | 0.0 (0.0,1.0) | 0.0 (0.0,1.0) | 0.365 | |
| Relief, frequency | 1.0 (1.0,1.0) | | 1.0 (1.0,1.0) | 1.0 (1.0,1.0) | 0.686 | |
| Annual eGFR decrease  [ml/ (min*1.73m^2^)] | 0.3 (-1.5,2.5) | | 0.0 (-1.6,2.1) | 0.4 (-1.1,2.9) | 0.264 | |
| **Present medication** |  | |  |  |  | |
| [Tripterygium](http://www.baidu.com/link?url=w78_K27LfSgr7lLWOcpb9CwAChkR2-1HjNJOkcz01Ro8Pq-742YMi-9wsrPvwFhHT9c_MECMuXzxvbYciWHFljo7rHTfO3s5m1ZBxSKoihG) | 48 (20.9) | | 27 (23.1) | 21 (18.6) | 0.402 | |
| Cyclosporine | 9 (3.9) | | 2 (1.7) | 7 (6.2) | 0.079 | |
| Tacrolimus | 7 (3.0) | | 4 (3.4) | 3 (2.7) | 0.736 | |
| Leflunomide | 11 (4.8) | | 6 (5.1) | 5 (4.4) | 0.803 | |
| Statin | 52 (22.6) | | 20 (17.1) | 28 (28.3) | 0.042 | |
| ACEI/ARB | 134 (58.3) | | 70 (59.8) | 64 (56.6) | 0.624 | |
| Other antihypertension drugs | 74 (32.7) | | 33 (28.9) | 41 (36.6) | 0.220 | |
| Laboratory findings, |  | |  |  |  | |
| FPG, mmol/L | 6.0 ± 1.2 | | 6.0 ± 1.2 | 5.9 ± 1.1 | 0.464 | |
| TG, mmol/L | 1.5 (1.1,2.2) | | 1.5 (1.1,2.2) | 1.4 (1.0,2.3) | 0.451 | |
| TyG index^b^ | 8.9 ± 0.6 | | 8.9 ± 0.6 | 8.8 ± 0.6 | 0.381 | |
| SCr, mg/dl | 0.9 (0.7,1.2) | | 0.9 (0.7,1.2) | 0.9 (0.6,1.4) | 0.867 | |
| eGFR, ml/min per 1.73m^2^ | 93.0 (58.0,108.0) | | 93.0 (63.1,108.8) | 93.0 (53.0,107.5) | 0.490 | |
| **Characteristics** | **No. ^a^** | | | | | **P Value** |
|  | **Total (n=230)** | **Low level of HDL-C(n = 117)** | | **High level of HDL-C(n = 113)** | |  |
| UA, μmol/L | 367.2 ± 106.7 | 374.0 ± 98.4 | | 360.2 ± 114.7 | | 0.327 |
| Alb, g/L | 42.7 (39.3,46.1) | 42.9 (39.2,46.4) | | 42.6 (39.6,45.8) | | 0.480 |
| UPR, g/24h | 0.6 (0.3,1.6) | 0.4 (0.3,1.4) | | 0.8 (0.3,1.8) | | 0.078 |

(continued)

a Percentages may not total 100 because of rounding; b TyG index:Triglyceride-glucose index = In (Triglyceride * Fasting blood glucose / 2) (unit: mg/d/dL); CR: complete remission; FPG: fasting plasma glucose; FSGS: focal segmental glomerulosclerosis; HDL-C: high-density lipoprotein cholesterol; INS: idiopathic nephrotic syndrome; NR: non-remission; PR: partial remission; FPG: Fasting plasma glucose; TG: Triglycerides; SCr: Serum creatinine; eGFR: Glomerular filtration rate estimated according to CKD-EPI Formula; Alb: Albumin;UPR:Urinary protein rate;
